# Individual and School Correlates of Adolescent Leisure Time Physical Activity in Quebec, Canada

**DOI:** 10.3390/ijerph15030412

**Published:** 2018-02-27

**Authors:** José Massougbodji, Alexandre Lebel, Philippe De Wals

**Affiliations:** 1 Department of Social and Preventive Medicine, School of Medicine, Laval University, Quebec City, QC G1V 0A6, Canada; Jose.Massougbodji@criucpq.ulaval.ca; 2 Evaluation Platform on Obesity Prevention, Quebec Heart and Lung Institute Research Center, Quebec City, QC G1V 4G5, Canada; alexandre.lebel@criucpq.ulaval.ca; 3 Graduate School of Land Management and Regional Planning, Laval University, Quebec City, QC G1K 9E5, Canada

**Keywords:** adolescents, built environment, geographic information system, leisure-time physical activity, school neighbourhoods

## Abstract

*Background*: Leisure time physical activity (LTPA) correlates have been mostly studied in relation to adolescents’ home neighbourhoods, but not so much in relation to the environment of their schools’ neighbourhoods. We sought to investigate how objective environmental measures of the schools’ vicinity are related to adolescents’ self-reported LTPA. *Methods*: Individual data from the Quebec High School Students Health Survey (QHSSHS) were matched with schools’ socioeconomic indicators, as well as geographic information system-based indicators of their built environments. Self-reported levels of LTPA during the school year were assessed according to intensity, frequency and index of energy expenditure. Associations per gender between covariates and LTPA were estimated using ordinal multilevel regression with multiple imputations. *Results*: Boys (21% of which were highly active) were more active than girls (16% of which were highly active) (*p* ≤ 0.01). The incremental variance between schools explained by the contextual variables in the final models was higher among girls (7.8%) than boys (2.8%). The number of parks or green spaces within 750 m around their schools was positively associated with student LTPA in both genders. *Conclusions*: The promotion of parks around schools seems to be an avenue to be strengthened.

## 1. Introduction

Promoting healthy lifestyle habits among individuals can slow down the progression of the obesity epidemic [1,2,3,4,5]. The earlier healthy lifestyles are taught, the more rooted and sustained they become [6]. Adolescence has been identified as a critical period that plays a key role in the development and persistence of overweight individuals and its comorbidities in adulthood [7]. One of the habits that helps to reduce the risk of obesity is the regular practice of adequate physical activity (PA) [8]. In developed countries, promoting leisure time physical activity (LTPA) among adolescents has been established as one of the most promising avenue in terms of potential health benefits [9]. Recent evidence showed that LTPA increased during childhood and decreased during adolescence with a decline more pronounced for girls than for boys [10,11,12].

PA correlates have been mostly studied in relation to the home neighbourhood but not so much in relation to the school environment [13,14]. However, a recent US study focusing on the location of adolescent LTPA practices, revealed that the proportion of their time used in moderate and vigorous physical activity (MVPA) was highest for locations near their homes and near their schools on school days [15]. Results also showed that girls had fewer MVPA minutes per day than boys in all locations, except near their schools.

In the last decade of publications on PA correlates, few studies have specifically targeted the dimension of LTPA among adolescents in studies, and their authors agree on several issues: LTPA should be addressed in a socio-ecological perspective, and future researches as well as public health recommendations should consider the environment in which this behaviour occurs [16,17].

The methods commonly used to characterise the environment around schools are considered subjective when they rely on individuals’ perceptions [18,19] or, as objective through the use of geographic information systems (GIS) [20,21]. Similarly, PA can be measured from individual self-reported data or objectively from devices such as accelerometers and pedometers. These latter devices, however, reach their limits when a specific domain of physical activity is concerned [22]. To our knowledge, no study has investigated LTPA among adolescents in relation to their school environments assessed through objective methods. The aims of this study were to: (a) describe self-reported levels of LTPA among boys and girls in the province of Quebec, Canada; (b) identify individual and contextual correlates with their levels of LTPA and (c) evaluate how students’ variations in LTPA could be explained by their schools’ neighbourhoods and their characteristics.

## 2. Materials and Methods

### 2.1. Study Design and Population Sample

Data used for this study was drawn from the Quebec High School Students Health Survey — QHSSHS (*Enquête Québécoise sur la Santé des Jeunes du Secondaire*—*EQSJS*), a cross-sectional student based survey which portrays their physical health and their lifestyle habits [23]. The target population for the HSQHSS was all the 430,000 students from 1st to 5th year of high school, who went to a public or private school, French or English speaking, in the fall of 2010. This population covers approximately 98.4% of all Quebec students enrolled in high school. A two-stage (schools and students) cluster sampling was used to constitute a sample of 63,196 students in 470 schools. The overall response rate was 88%. Data collection was conducted using an anonymous self-administered questionnaire on a netbook during a class period, in the presence of two interviewers and the teacher, and arrangements were made to ensure students’ responses remained confidential. Further details about the survey are available in a previous publication [23].

The analyses for this study were restricted to students who attended schools for which complete buffer data were available, which represented 457 of the 470 schools in the initial QHSSHS sample. These 457 schools included an overall number of 62,015 students. Since some schools were exclusively attended by students of the same gender, these 457 schools were grouped into 452 and 442 schools, attended respectively by girls and boys. An additional 163 students (63 girls and 100 boys) were excluded because of missing data on their practice of LTPA. The final analyses involved 31,111 girls from 452 schools and 30,749 boys from 442 schools. A total of 61,860 students were thus included in our analysis. Since information of the student’s school geolocation was available in the QHSSHS, student data was matched with data relating to their school, provided by two other databases: the environmental database of the characteristics of the built environment around schools in the whole province of Quebec, compiled by the Statistical Institute of Quebec and the Ministry of Education, Recreation and Sports’ database on deprivation indexes of public schools in Quebec. The students’ home neighbourhoods could not be matched with their data since students’ residential addresses were concealed from us on the grounds of confidentiality.

### 2.2. Outcome

Self-reported levels of LTPA e.g., PA done during free time, at home, at school or elsewhere was our primary outcome. Physical activity performed as part of the school physical education program, related to active commuting (school, work, etc.), or performed in the context of any paid employment was excluded from our analysis. In the QHSSHS questionnaire, a generic list of 5 leisure activities (sports, outdoors, fitness, dancing or just walking) was presented to the students as well as five questions related to LTPA: (1) “*During the school year, did you do these activities?*” (2) “*Usually, during the school year, do you do these activities every week?*” (3) “*Usually during the school year, how many days per week do you do these activities?*” (4) “*In a typical day of the school year, how long do you do this kind of activity?*” (5) “*Most often, when you are doing such activities, is your level of physical effort very low, low, moderate or high?*” Those questions helped build a composite indicator that measure global LTPA levels. This indicator derived from the World Health Organization standards for the weekly amount of PA and previously used in Quebec for many years to monitor PA, was considered for each of the predefined leisure activities, the intensity (Metabolic Equivalents of Task (METs)), the frequency (days per week) and the index of energy expenditure (kcal/kg/week). In our study, LTPA levels were defined on an ordinal scale: “*Highly active*” which corresponds to the WHO’s international recommendation for energy spending for 5 to 17-year-olds, “*Moderately active*”, “*Slightly active*” and “*Sedentary*”. The WHO’s international recommendation for energy spending for 5 to 17-year-olds corresponds to at least 60 min/day, of moderate to high intensive activity, every day, which is the equivalent of an expenditure volume of at least 30 kcal/kg^−1^ week^−1^, reached with a frequency of five or more practices per day/week and an intensity of more than 3 MET. Further details on codification, criteria and algorithms for the characterisation of the levels of practice are described more explicitly elsewhere [24] and the extended definitions of LTPA levels are provided in Supplementary Materials Parts 1 and 2.

### 2.3. Individual Characteristics

Data on students’ characteristics were self-reported and were anthropometric (weight status), demographic (educational level, educational curriculum), socioeconomic (family situation, parents’ employment status), behavioural (smoking, alcohol and illicit drug consumption) and psychosocial (perception of their own health, satisfaction with their body image). More details of these individual characteristics and their assessment are available in Supplementary Materials Part 2.

### 2.4. Contextual Characteristics

Variables related to built environment around schools during the survey were GIS-based and derived from disaggregated data from several official databases pertaining to years 2007 to 2011. They were selected on the basis of a previous study reviewing the robustness of commonly used indicators to assess the relationship between attributes of built environment and healthy lifestyles [25]. These variables have already been used in other studies in Quebec [26]. For greater accuracy, there were computed at the level of school buildings rather than the official school address and the location of the school buildings was geocoded using the complete civic address. These indicators are assumed to be robust given that: (1) they were calculated considering the road network distance (excluding highways), which is known to be more predictive of human commuting than the Euclidean distance; (2) they take into account informal transportation networks such as pedestrian trails; and (3) they were computed using sausage network buffers, which consisted in a maximization of the road-network distance and a termination by the Euclidean distance. Beyond its ease of use and reproductibility, this method has the advantage of minimizing distortions due to a low road network density, most often encountered in rural areas [27]. Buffers centred on the school buildings within five threshold distances (250; 500; 750; 1000 and 1500 m) were used to calculate geographic features [28,29]. These included measures of spatial accessibility (availability and proximity) to parks, green spaces or leisure amenities, a vegetation index, information on urban density assessed with density-based indicators (i.e., residential density, land use ratio, intersection density) [9,30,31], and the presence of a highway in the school surroundings [13]. The availability of parks, green spaces and leisure amenities were assessed by their count within each buffer and road-network distance-based proximity metrics were calculated to assess their geographic accessibility. A walkability index was computed from the residential density, the land use ratio and the intersection density. Kernel density was used to estimate intersection density. More precise definitions of these variables and key terms are provided in Supplementary Materials Part 3.

The school’s level of deprivation was also considered. The information, provided annually for schools across the province, was assessed by the school socio-economic index (SSEI), which has a social and an economic dimension based on the home addresses of parents of children attending the school. The social dimension is derived from the mean level of education of mothers with a child or children aged 0 to 18 in the dissemination area the student is living, and an economic dimension evaluating parents’ employment statuses [32]. Data on dissemination areas were derived from the 2006 Canadian census. SSEI corresponds to the average of all students' SSEI. The index was adjusted for the school year 2010–2011. SSEIs were grouped into three categories corresponding to the tertiles of its distribution. Moreover, since this index was available for public schools only, we created an additional category to regroup all private schools assuming that private schools would be the least deprived of all schools.

### 2.5. Statistical Analysis

Considering the ordinal nature of the main outcome variable (LTPA) and the nested structure of the data (students in schools), we ran hierarchical generalised linear models fitted with the PROC GLIMMIX with fixed slopes and cumulative logit link function, to examine the associations between the level of LTPA and its correlates. We performed two separate models per gender. An assessment of missing data on individual variables revealed that the highest rates were less than 3%, except for BMI (10.5%) and satisfaction with body image (50%). The question of body image was only asked to half of the students because of the length of the questionnaires and the diversity of themes explored by the QHSSHS. Multiple imputations were performed to handle missing data on independent variables. Twelve datasets were imputed for continuous and discrete variables [33,34], with the Markov chain Monte Carlo statement (6000 chain iterations). For each of the contextual variables which were quantitative, the literature was searched for their usual categorisation. When no proper categorisation was found, the variable was coded in groups, according to its quartiles, tertiles and median. Then, a bivariate regression was performed with each of these categorisations, including the variable left as continuous. The categorisation that was retained was the one with the best explained deviance. Since built environment indicators were calculated for different threshold distances (250; 500; 750; 1000 and 1500 m), a preliminary analysis was performed using several bivariate multilevel type regressions to identify for each environmental variable, the threshold explaining best the level of student LTPA. The reason we used the walkability index in the main analysis was because of its consistent relationship with adolescent PA in the literature. We also examined the associations between individual characteristics and the outcome in bivariate regression models and only variables that were significantly associated with LTPA were included in the multivariate models. After ensuring the absence of multicollinearity issues, we ran multivariate models using a backward method such as selection criteria, the *p*-value and the Bayesian Information Criterion (BIC). Measures of association presented in our models are cumulative odds ratios. We’ve tested cross-level interactions between contextual variables and known moderators such as the education level and BMI. To build our models, statistical significance was defined as *p*-values < 0.10 for bivariate analyses and *p*-value < 0.05 for multivariate analysis (2-sided test).

To quantify the extent to which the variables in the final models explained the neighbourhood effects, we ran the same models with the same variables, but this time, in an incremental way. We tested three gradual model specifications: an ‘empty’ one without correlates to detect the existence of a possible contextual effect, a second one with individual variables and a third one with both individual and contextual variables. Sensitive analyses consisted of: (1) running analyses restricted to cases without any missing information (2) replacing the walkability index by its composite variables (e.g., residential density, land use ratio, intersection density and destination density). All analyses were performed in the Statistical Analysis System (SAS Institute Inc., Cary, NC, USA, version 9.3).

## 3. Results

### 3.1. Characteristics of the Study Population

Overall, 16% of students reached the recommended level (highly active) of LTPA during the school year. In contrast, over a third (34%) was sedentary. Approximately 50% of the students reported being ‘slightly active’ or ‘moderately active’. However, as shown in Table 1 which lists the students’ general characteristics per gender, boys (21% of which were highly active) were more active than girls (16% of which were highly active) (*p* ≤ 0.01).

Because they raised multicollinearity issues, educational level was preferred to age for the rest of the analyses, since most of the LTPAs explored in the QHSSHS were done with peers. There were more girls attending a school teaching the general curriculum (95%) than boys (91.4%). When they were not satisfied with their body image, girls wanted to lose weight while boys wanted to gain some. Girls tended to report the willingness to lose weight whereas boys desired to gain weight (*p* ≤ 0.01). More boys had an excellent or very good perception of their own health (75% vs. 67% for girls). More boys also reported having regular consumption of alcohol in their lives (13% vs. 10% among girls).

### 3.2. Quantifying the Influence of the School’s Neighbourhood

As shown in Table 2, individual correlates explained more of the variance between schools in LTPA among boys (21.8%) than girls (23.9%). However, the incremental variance explained by the contextual variables in the final models is higher among girls (7.8%) than boys (2.8%). Overall, all the individual and contextual correlates in the final models explained the variance in LTPA for girls and boys, at 23.9% and 24.6% respectively.

### 3.3. LTPA Correlates

The results of the multivariate analysis of factors associated with boys’ and girls’ LTPA are presented in Table 3. For simplicity, only significant associations are reported. In both genders, greater odds of LTPA were significantly associated with receiving a general curriculum education, being of normal weight, living in a two-parent family, having both parents in employment, and perceiving oneself in good general health. However, gender specific results were noted: among girls, a regular consumption of illicit drugs was associated with lower levels of LTPA. Among boys, any dissatisfaction with their body image was associated with fewer odds of LTPA. Also, a positive association between the overweight status with the LTPA (Odd ratio OR = 1.09; 95% confidence interval CI = 1.02, 1.17) was found. Finally, a discordant effect of the education level on LTPA per gender was found: being of higher education level was associated with higher levels of LTPA, while the opposite trend was observed for girls.

Very few contextual variables were significantly associated with student LTPA in the multivariate models and their gender specificity was more pronounced: among girls, it appeared that going to a private school, compared to the most advantaged public schools, was associated with an increase of LTPA. We also found that among girls, there existed a cross-level interaction between the number of parks or green spaces within 750 m of their schools and their education level, showing that a decrease of LTPA along with the education level is more intense among girls with a low number of parks around their schools (OR = 0.89; 95% CI= 0.81, 0.98). Boys also had their level of LTPA positively associated with the number of parks or green spaces within 750 m around their schools, irrespective of their education level.

## 4. Discussion

In our analysis, differences in the LTPA levels by gender and the individual factors associated with these levels in girls and boys are in line with our expectations and results of other studies. Although a decline in PA is usually observed with aging in adolescence [12,35], in our study, boys of Grades 9, 10 and 11 seemed to be more active than those who were younger in Grades 7 and 8. This result is consistent with those of a Danish study which compared the objectively measured levels of the adolescent PA according to gender and age [36]. The authors reported that adolescent boys spent a larger proportion of their MVPA outdoors during leisure in school grounds and other places (sports facilities or shopping centres around school and home), while boys who were children had more outdoor MVPA only during recess and school hours. The same may apply in Quebec where older boys would feel more free to engage in LTPA beyond locations under supervision or without major time constraints.

As expected, low levels of LTPA were found among obese and underweighted boys. However, the overweight status was positively associated with LTPA. This could be due to boys’ willingness to be more active to reduce their weight or, to a social desirability bias in self-reporting their LTPA levels. Also, the weight status in our study has been defined by the BMI indicator which may be inadequate to distinguish between the adipose and the muscular nature of an overweight condition [37].

As found in other studies [38,39], LTPA contextual correlates were different per gender. The positive association between LTPA and school socio-economic status (SES) was more pronounced for girls than boys. As expected, boys and girls in private schools had the highest LTPA levels and this could be explained by a greater valorisation of PA in families sending their children to private schools and/or a more supportive environment for LTPA through internal amenities in these schools. In public schools, the school deprivation index was positively associated with LTPA in girls but not in boys. In another prospective study in Ontario, it was observed that participation in active free play increases faster over time for those living in high SES neighbourhoods relative to those living in the lowest SES neighbourhood in girls but not in boys [24]. An explanation proposed by the authors of this study is that girls living in low-income neighbourhoods are more involved in domestic activities and have safety concerns for outdoors activities [24]. In our study, the school deprivation index is computed from information on each student household SES, and thus, is correlated with the home neighbourhood SES.

The role of parks and green spaces merits special attention. More parks in the school surroundings was associated with an increasing level of LTPA among boys and particularly among older girls. Although our study design does not allow any inference on a causal mechanism in this association, it may be well that parks play a role in facilitating out of school LTPA. We also made further investigations to see whether the schools’ levels of deprivation were correlated to the number of parks around them but this was not the case. As green spaces and leisure parks may contribute to the improvement of health in different ways, it would be important to assess whether new local urban developments including leisure parks and green spaces are associated with an increase in LTPA of residents and school students. If this is the case, such interventions should preferably focus on socially disadvantaged areas in which LTPA levels are the lowest as found in our study.

We also aimed to quantify the extent to which the school environment could explain the variance between schools in student LTPA. Among girls, contextual variables explained approximately 10% of the variance between schools and the proportion was only 3% for boys. These proportions are consistent with those reported in another study in the US (2.4% in boys and 4.5% in girls) which concludes that measured individual variables are more predictive than measured contextual variables in explaining physical activity among adolescents [17]. This doesn’t mean that environmental interventions aiming to promote both healthy nutrition and physical activity should be discarded. Most of the individual variables found to be associated with LTPA in our study are not or not easily modifiable (i.e., gender, age, social status, scholar curriculum) whereas our capacity to modify the environment is far greater. The assessment of the impact of a coordinated school-based program that incorporated recommendations for school-based healthy eating programs and the promotion of physical activity was associated with healthier diets, increased physical activities and a lower obesity rate [40]. Obviously, the effect of environmental changes in obesity is not direct and mediated by changes in the diet and the level of physical activity at individual levels. In addition, a healthy weight is also a factor in favour of increased physical activity as observed in our study. The complex relationship between environmental and individual predictors of LTPA in adolescents should ideally be measured in prospective studies as should the use of structural models incorporating direct and indirect causal pathways [41].

A limitation in our study is the way environmental variables were selected and measured. They were not specifically related to the main outcome which is the level of physical activity during leisure time. In a study in Portugal [26], it was found that environmental factors were predictive of the non-organised LTPA but not of organised LTPA. This partition could not be made from data collected in our survey. Also, activities occurring during the school curriculum and during active commuting were not measured. Ideally, a global and objective measure of all physical activities including the duration and intensity which may be collected through the use of instruments such as accelerometers should be made in connection with a questionnaire regarding the nature and context of the physical activity. In our study, contextual factors identified in environmental databases were measured and related to subjective measurements of LTPA but not the subjective perception of environmental influences which use is advocated by several authors [17,21,22,42]. Also, the absence of information in our study on an important psychological factor which is the self-efficacy regarding physical activity is a limitation. This factor has been found to be a key moderator of the environmental influences on the physical activity of adolescents in several studies [18,43,44,45]. In the EQSIJS survey, there was one general question of the self-efficacy but not related to a particular behaviour.

In our study, the only geo-localised variable was the school, and all environmental factors that were included in the analysis were those related to schools. In Quebec, the selection of a secondary school is mainly decided by parents according to a large variety of criteria including proximity. Information on the location of students’ homes was not available. It may be that environmental influences in the home neighbourhood are a more important determinant of adolescent LTPA than environmental influences in the school environment knowing that more time is spent in the school than in its vicinity. This significant limitation may also contribute to explain the low rates of LTPA observed in girls who are likely to prefer to exercise at home rather than outdoors. In future surveys, it would be important to collect geo-localised data on the students’ homes to be able to refine the analysis of environmental influences. In addition, intra-home environmental influences are other important determinants of adolescent behaviours which were not measured in our study [18,30,36,46].

Study strengths include the large sample size, the representative nature of the sample for secondary school students in Quebec and an objective assessment of the physical environment near schools within 5 buffer zones. Limitations relate to the cross-sectional design that precludes any causal relation between LTPA and its correlates, the use of self-reported data of LTPA which is prone to memorisation bias, as students were asked to recall a one-year period, and the questionnaire’s inability to consider seasonal variations of LTPA.

The objective of the QHSSHS survey was to portray adolescents’ physical and mental health and some selected lifestyle habits like PA. The survey wasn’t designed to measure all the components of PA and their predictors. In the future, questions on commuting and activity during school hours should be included. Also, information on the residential addresses which was collected, but not made available to researchers on the grounds of confidentiality, is a major issue. The scientific usefulness of population-based data would be considerably enhanced if their connection to environmental databases had been made on both the residential and school addresses. This would allow us to consider adolescents’ activity spaces and their mobility and thus, to have a dynamic approach to assessing the physical environment exposure [46].

In summary, our recommendations for future LTPA correlate studies are more detailed and comprehensive measures of levels of LTPA when it is self-reported, a better definition of the context of LTPA per type, time and location, and the use of both objective and perceived measures of built environment. More longitudinal studies are also needed.

## 5. Conclusions

Although we found a relatively small influence of the school neighbourhood on adolescent LTPA, the promotion of parks seems to be an avenue to be strengthened. In addition, more emphasis may be placed on the design of policies and interventions targeting psychosocial determinants of adolescent girls’ LTPA from disadvantaged backgrounds, in the middle and high school age ranges.

## Figures and Tables

**Table 1:** General Characteristics of the Students Involved in the QHSSHS, Quebec, 2010–2011.

Characteristics		Girls (%)	Boys (%)
		*n* = 31,111	*n* = 30,749
LTPA levels	Highly active	10.4	21.1
	Moderately active	22.7	28.2
	Slightly active	28.3	20.6
	Sedentary	38.6	30.1
Curriculum	General curriculum	95.0	91.4
	Other types of curriculum	5.0	8.6
	Missing data		
Educational level	Grades 7 and 8 ^a^	40	58.2
	Grades 9, 10 and 11	60	41.8
	Missing data		
Satisfaction with body image	Satisfaction	25.8	25.7
	Desire of slight loss	16.4	9.5
	Desire of heavy loss	4.4	2.6
	Desire of slight gain	3.7	10.3
	Desire of heavy gain	0.0	1.9
	Missing data	49.7	50.0
Family situation	Two-parent family	60.6	62.7
	Stepparent family	14.6	10.3
	Single parent family	12.5	13.9
	Shared custody	10.5	11.6
	Others	1.8	1.4
	Missing data	<0.01	0.1
Family employment status			
	Both parents employed	69.6	69.2
	A single parent employed	19.1	19.1
	Both parents unemployed	3.4	3.2
	Missing data	8.0	8.5
Perceived health	Excellent or very good	67.3	74.8
	Good	27.7	21.4
	Fair or poor	4.9	3.7
	Missing data	<0.01	0.1
Weight status	Underweight	10.9	7.3
	Normal weight	62.5	59.7
	Overweight without obesity	9.6	15.6
	Obesity	5.1	6.9
	Missing data	11.9	10.5
Smoking status ^b^	Current smokers	6.7	6.9
	Beginning smokers	3.9	3.2
	Non-smokers	87.6	88.2
	Missing data	1.7	1.7
Regular^c^ consumption of alcohol	No	90.2	86.3
	Yes	9.7	13.4
	Missing data	0.1	0.3
Regular^c^ consumption of illicit drugs	No	89.5	86.9
	Yes	10.4	13.0
	Missing data	0.1	<0.01

Abbreviations: HSQHSS, Quebec High School Students Health Survey; LTPA, Leisure-Time Physical Activity. ^a^ Grade 7 students were mostly under 13 years old; Grade 8 students were mostly between 13 and 14 years old; Grade 9 students were mostly between 14 and 15 years old; Grade 10 students were mostly between 15 and 16 years old; Grade 11 students were mostly between 16 and 17 years old; ^b^ Current smokers = at least 100 cigarettes in their lifetime and smoking cigarettes every day (daily) or less often than every day (occasional) during the last 30 days; Beginning smokers = less than 100 cigarettes in their lifetime and who smoked in the last 30 days; Non-smokers = former smokers (at least 100 cigarettes in their lifetime but didn’t smoke in the last 30 days) or former experimenters (less than 100 cigarettes in their lifetime but didn’t smoke in the last 30 days) or definitely non-smokers (never smoked or less than a whole cigarette in their lifetime); ^c^ At least once a week for at least a month.

**Table 2:** Random Effects of School Neighbourhood on Leisure-Time Physical Activity Among Adolescents Involved in the QHSSHS, Quebec, 2010–2011 (with Multiple Imputation).

Models	Girls		Boys	
	Variance (SE)	Proportional Change in Variance (%)	Variance (SE)	Proportional Change in Variance (%)
Empty model ^a^	0.25 (0.02) *		0.17 (0.02) *	
Model with only individual correlates ^b^	0.21 (0.02) *	−16.11	0.13 (0.01) *	−21.75
Model with individual and contextual correlates ^c^	0.20 (0.02) *	−7.76	0.13 (0.01) *	−2.80

Abbreviations: HSQHSS, Quebec High School Students Health Survey; SE, Standard error. ^a^ Model with no correlates; crude model; ^b^ Model α adjusted for all relevant individual correlates in the parsimonious model of LTPA; ^c^ Model β adjusted for all relevant contextual correlates in the parsimonious model of LTPA; * = *p* < 0.001.

**Table 3:** Correlates of Leisure-Time Physical Activity Among Adolescents Involved in the QHSSHS, Quebec, 2010–2011 (with Multiple Imputation).

Parameter		Models of Leisure-Time Physical Activity			
		Girls		Boys	
		Level 1 (Students): *n* = 31,111; Level 2 (Schools): *n* = 452		Level 1 (Students): *n* = 30,741; Level 2 (Schools): *n* = 442	
		OR*^α^*	95% CI	OR	95% CI
*Student-level characteristics*					
Curriculum (vs. General curriculum)	Other types of curriculum	**0.56**	**0.50, 0.63**	**0.56**	**0.51, 0.61**
Education level (vs. Grades 7 and 8)	Grades 9. 10 and 11	0.95	0.89, 1.02	**1.13**	**1.08, 1.19**
Satisfaction with body image (vs. Satisfaction)	Desire of slight loss	1.02	0.97, 1.07	**0.85**	**0.80, 0.90**
	Desire of heavy loss	0.96	0.87, 1.05	**0.69**	**0.61, 0.79**
	Desire of slight gain	**0.77**	**0.70, 0.86**	**0.88**	**0.83, 0.94**
	Desire of heavy gain	0.97	0.61, 1.56	**0.65**	**0.56, 0.76**
Weight status (vs. Normal weight)	Underweight	**0.82**	**0.76, 0.89**	**0.59**	**0.54, 0.64**
	Overweight without obesity	0.93	0.86, 1.02	**1.09**	**1.02, 1.17**
	Obesity	**0.81**	**0.74, 0.89**	**0.84**	**0.75, 0.92**
Family situation (vs. Two-parent family)	Stepparent family	**0.87**	**0.81, 0.93**	**0.89**	**0.83, 0.96**
	Single parent family	**0.88**	**0.83, 0.94**	**0.82**	**0.77, 0.87**
	Shared custody	1.08	1.00, 1.15	0.98	0.92, 1.05
	Others	0.89	0.75, 1.05	1.02	0.84, 1.24
Family employment status (vs. Both parents employed)	A single parent employed	**0.78**	**0.73, 0.82**	**0.87**	**0.83, 0.92**
	Both parents unemployed	**0.65**	**0.57, 0.75**	**0.65**	**0.57, 0.75**
Perceived health (vs. Excellent or very good)	Good	**0.54**	**0.52, 0.57**	**0.41**	**0.39, 0.43**
	Fair or poor	**0.36**	**0.32, 0.40**	**0.29**	**0.25, 0.32**
Regular * consumption of alcohol (vs. No)	Yes	**1.17**	**1.08, 1.27**	**1.47**	**1.38, 1.58**
Smoking status (vs. Non-smokers)	Current smokers	**0.86**	**0.76, 0.95**	**0.83**	**0.76, 0.92**
	Beginning smokers	0.96	0.85, 1.07	0.94	0.83, 1.05
Regular ^a^ consumption of illicit drugs (vs. No)	Yes	**0.81**	**0.75, 0.87**	0.98	0.91, 1.06
*School-level characteristics*					
Schools deprivation index (vs. Most favoured public schools tertile)	Moderately favoured public schools tertile	0.91	0.79, 1.03	1.02	0.91, 1.13
	Less favoured public schools tertile	0.97	0.85, 1.11	1.03	0.92, 1.16
	Private schools	**1.27**	**1.10, 1.46**	**1.16**	**1.02, 1.31**
Number of parks or green spaces within 750 m around the school (vs. High = 2 or more)	1 or 0 (low)	0.94	0.84, 1.05	**0.90**	**0.82, 0.97**
*Cross level interactions*					
Low number of parks or green spaces × Education level ^b^		**0.89**	**0.81, 0.98**	1.07	0.98, 1.17

Abbreviations: HSQHSS, Quebec High School Students Health Survey; OR, Odds ratio (In our analysis, we ran multinomial logistic regression. Therefore, ORs are cumulative odds ratios, which are an average of the 3 logistic comparisons of LTPA levels: ‘Sedentary’ vs. others, ‘Sedentary’ or ‘Slightly active’ vs. ‘Moderately active’ or ‘Highly active’ and other vs or ‘Highly active’); 95% CI, 95% confidence interval. ^a^ at least once a week for at least a month. ^b^ Reference category: high number of parks or green spaces and lower education level; * *p* < 0.001.

## Supplementary Materials

Supplementary Materials are available online at http://www.mdpi.com/1660-4601/15/3/412/s1.
