# Supplementary material for: Individual and School Correlates of Adolescent Leisure Time Physical Activity in Quebec, Canada
Source: Int J Environ Res Public Health. 2018 Feb 27;15(3):412. doi: 10.3390/ijerph15030412 (PMC5876957; doi:10.3390/ijerph15030412)
Supplement: Supplementary file 1 [file ijerph-15-00412-s001.pdf]

International Journal of
Environmental Research and Public Health

MDPI

Supplementary Materials

Supplementary Material 1: Method of Calculation of the Index of Energy Expenditure (IEE) and cHaracterisation of Students' Level of LTPA (Adapted from Pica L. [23])

A. Questions related to LTPA in the QHSSHS questionnaire

The following questions are about leisure time physical activities at home, at school or elsewhere: sports, outdoors, fitness, dancing or just walking.

These can be activities practiced in an organized context (with a monitor, a coach or other responsible person) or unorganized (alone or with other people). Special program activities (sports and studies, dance and studies or other programs) must also be reported in your answers.

Q1 During the school year, did you do these activities?

☐ Yes

☐ No

Q2 Usually, during the school year, do you do these activities every week?

☐ Yes

☐ No

Q3 Usually during the school year, how many days per week do you do these activities?

☐ 1 day per week

☐ 2 days per week

☐ 3 days per week

☐ 4 days per week

☐ 5 days per week

☐ 6 days per week

☐ 7 days per week

Q4 In a typical day of the school year, how long do you do this kind of activity?

☐ Less than 10 min

☐ 10 to 19 min

☐ 20 to 39 min

☐ 40 to 59 min

☐ 1h to 1h 29 min

☐ 1h 30 min to 1h 59 min

☐ More than 2 hours

Q5 Most often, when you are doing such activities, your level of physical effort is:

☐ Very low (breathing and heartbeats very little modified)

☐ Low (breathing and heartbeats a little faster)

☐ Moderate (breathing and heartbeats fast enough)

☐ High (breathing and very fast heartbeat

B. Determination of values used to calculate the IEE

1. Physical activity frequency

The value given for the answers to questions Q2 and Q3 (frequency per week) correspond to the choice of the respondent (example: 2 days a week = 2)

2. Physical activity duration

Table S1 specifies the duration assigned according to the choice of answers to questions Q2 and Q4 (time per day). The value "0" hour is assigned for the first choice of answer (less than 10 minutes), according to the present recommendations. Also this choice refers to the total duration in a typical day and not to a particular session throughout the day. So this choice answer (less than 10 minutes) automatically classifies the individual in the sedentary level. The central value of the time interval is assigned for the other five choices.

Table S1. Assigned duration, to questions Q2 and Q4, according to the answer choice

Answer choice	Duration (hours)
Less than 10 min	0
10 to 19 min	0.25
20 to 39 min	0.50
40 to 59 min	0.83
1h to 1h 29 min	1.25
1h 30 min to 1h 59 min	1.75
More than 2 hours	2.25

3. Physical activity intensity

For the level of physical effort (intensity), a value, in METs, is attributed to each of the response options (Table S2).

Table S2. Assigned intensity in METs, to question Q5, according to the answer choice

Answer choice	Duration (hours)
Very low	2.2
Low	4.5

Moderate	6.8
High	9.4

C. Calculation of the IEE

The IEE is obtained in the following way: frequency x duration x intensity.

Example: 5 days / week x 0.83 h / day x 4 METs (4 kcal /kg / hour*) = 16.6 kcal / kg / week (achieved IEE).

*: Based on the following approximation: 1 MET ≈ 1 kcal / kg / hour.

D. Characterisation of students' level of LTPA

So, for this example (intensity of 4 METs, frequency 5 days / week and IEE of 16.6 kcal / kg / week), the level physical activity would be "moderately active" (see Table S3). Finally, the sedentary level is automatically assigned if the answer choice is "Less than 10 minutes" to question Q4.

Table S3. Definition of LTPA levels

LTPA level	Intensity (METs)	Frequency (days/week)	index of energy expenditure (kcal/kg/week)
Active	≥ 3	≥ 5	≥ 30
Moderately active	≥ 3	≥ 3	≥ 15
Mildly active	≥ 3	≥ 3	≥ 7.5
Barely active	Any	≥ 1	≥ 0
Sedentary	Any	< 1	Any

The three criteria (intensity, frequency and IDE) must be respected to be classified at a given level. More details are provided in the following references: [23–24].

Supplementary Material 2: Definition of Individual Variables

a) Levels of LTPA

• *'Active'*: The required volume (≥30 kcal·kg^{-1}·week^{-1}) must be reached with a frequency of five or more practices per day/week and an intensity of more than 3 MET. This volume (≥30 kcal·kg^{-1}·week^{-1}) is the equivalent of the WHO's international recommendation for energy spending for 5 to 17-year-olds (e.g. ≥60 minutes/day, of moderate to high intensive activity, every day).

• *'Moderately active'*: A volume of ≥15 kcal·kg^{-1}·week^{-1} and < 30 kcal·kg^{-1}·week^{-1}, reached with a frequency of three or more practices per day/week and an intensity of more than 3 MET.

• *'Mildly active' and 'barely active':* A volume of < 15 kcal·kg^{-1}·week^{-1}, reached with a frequency of at least one practice per day/week with no minimal intensity requirements

• *'Sedentary'*: Less than one practice per week or no activity whatsoever. This category also includes students reporting less than 10 minutes of PA in a typical day. Nonetheless only a small proportion of students belong to this group (1.1%).

In our study, the level "*Active*" was renamed "*Highly active*". Also, the categories "*barely active*" and "*mildly active*" were combined in one "*Slightly active*" category since taken separately, they are clinically meaningless.

b) Educational curriculum:

Information about the type of the educational curriculum (general curriculum versus other curriculum) was also sought from the person in charge of the school, for each selected class. The other types of curriculum are mainly for adolescents with learning disabilities.

c) Satisfaction with body image:

A valid and usable visual tool was used to assess their current and desired physical appearances [2–3]. This tool was a seven-unit gradual scale ranging from very thin to very strong body shape. Then an index of satisfaction with their body appearance was derived by making the difference between the current and the desired appearance. The satisfaction index included five categories: (1) 'satisfied' when the student selected the same shape twice, meaning he/she desires to maintain his/her appearance; (2) 'desire for a slight weight loss' when he/she chose a slimmer figure as his/her desired appearance with a gap of 1 unit; (3) 'desire for a major weight loss' when his/her desired appearance was slimmer with a gap ≤ 2 units. (4) 'desire for a slight weight gain' when his/her desired appearance was larger with a gap of 1 unit; (5) and 'desire for a major weight gain' when his/her desired appearance was larger with a gap ≥ 2 unit.

d) Family situation:

Students were asked to report their type of family life: (1) two-parent family (students living with both biological or adoptive parents); (2) stepfamily (students living with their mother or father and the mother's/father's spouse); (3) single parent family (students living with their mother or father only); (4) shared custody (students whose parents are separate and equally sharing their time with them); (5) and special situations (tutoring, family or foster home; roommates, independent, etc.).

e) Family employment status:

The parents' employment status was chosen to be an indicator of the economic status of students' families. This indicator was defined in three ways: (1) both parents at work; (2) only one parent working; (3) and both parents unemployed.

f) Clustered harmful behaviours:

Several harmful behaviours to the adolescents' health such as smoking, alcohol and illicit drug consumption were also collected in the QHSSHS. The smoking status indicator was constructed according to the number of cigarettes smoked during life and the frequency of consumption reported by students during the thirty days preceding the survey. A typology of alcohol and illicit drugs also based on frequency of use reported by students was used. Finally, information on the perception of the students' health as the only psychosocial-level variable was also composited.

Supplementary Material 3: Definitions and Built Environment Variables

a) Road network distance: Shortest distance on the road network between the school building and one or more locations.
b) Euclidean distance: Shortest distance 'as the crow flies' between the school and one or more locations
c) Buffer zone: Area within a distance (Euclidean or reticular) from a predetermined starting location; in this case, the school building.
d) Spatial Join: Operation to join the attributes of a group of entities (e.g. the number of housing residential units) with another group of entities (e.g. school buffer zones) based on a spatial relationship (e.g. superposition).

e) Residential density index: Spatial join between residential units and the buffer zone. Summation of the number of dwellings and number of dwellings per km2 of the buffer zone area.

f) Land uses ratio: Considers five types of land use: residential, commercial, industrial, recreational, cultural and service. Spatial join between duty units and the buffer zone. Summation of the occupied areas by each type of use and calculation of the entropy index

g) Intersection density index: assessment by kernel density. Extraction and average density value of the overlapping cells by the buffer zone.

h) Destination Density Index: Total property assessment units connected the duties of manufacturing industry, residential, commercial, services and cultural, divided by the number of square kilometres of the area buffer.

i) Walkability index: Sum of the Z-scores of the four components of the index: intersection density index, land uses ratio, residential density index and destination density Index.

j) Normalised Difference Vegetation index (NDVI) Standardised measure derived from satellite imagery (Landsat), represented by values expressing the presence of plant cover in the neighbourhood of the school buildings. The range of values for this measure is from 0 to 200, 0 expressing total lack of vegetation and 200 as being covered by a dense forest.

Table S4. Correlates of Leisure-Time Physical Activity Among Adolescents Involved in the QHSSHS, Quebec, 2010–2011 (Complete Case Analysis)

**Parameter**		**Models of Leisure-Time Physical Activity**			
		Girls		Boys	
		Level 1 (students): *n* = 12,682; Level 2 (schools): *n* = 446		Level 1 (students): n = 12,673; Level 2 (schools): *n* = 439	
		Parsimonious model**		Parsimonious model	
		OR	95% CI	OR	95% CI
Fixed effects					
Student-level characteristics					
Curriculum (vs. General curriculum)	Other types of curriculum	**0.59**	**0.49, 0.72**	**0.59**	**0.51, 0.69**
Education level (vs. Grades 7and 8)	Grade 9. 10 and 11	**0.84**	**0.77, 0.90**	**1.09**	**1.01, 1.18**
Satisfaction with body image (vs. Satisfaction)	Desire of slight loss	1.04	0.96, 1.12	**0.78**	**0.71, 0.86**
	Desire of heavy loss	1.09	0.95, 1.25	**0.66**	**0.56, 0.78**
	Desire of slight gain	**0.79**	**0.69, 0.91**	**0.85**	**0.78, 0.93**
	Desire of heavy gain	1.17	0.70, 1.98	**0.63**	**0.53, 0.76**
Weight status (vs. Normal weight)	underweight	**0.85**	**0.76, 0.95**	**0.57**	**0.50, 0.65**
	Overweight without obesity	**0.81**	**0.72, 0.91**	**1.15**	**1.05, 1.27**
	Obesity	**0.80**	**0.69, 0.93**	**0.82**	**0.71, 0.95**

Table S1. *Cont.*

	Step-parent family	**0.84**	**0.75, 0.93**	0.94	0.84, 1.06

Family situation (vs. Two-parent family)	Single parent family	0.99	0.89, 1.10	0.90	0.81, 1.00
	Shared custody	1.07	0.96, 1.19	0.98	0.86, 1.06
	Others	0.78	0.58, 1.04	0.92	0.66, 1.29
Family employment status (vs. Both parents employed)	A single parent employed	**0.81**	**0.74, 0.88**	**0.85**	**0.79, 0.93**
	Both parents unemployed	**0.56**	**0.45, 0.68**	**0.78**	**0.64, 0.95**
Perceived health (vs. Excellent or very good)	Good	**0.53**	**0.49, 0.57**	**0.41**	**0.38, 0.43**
	Fair or poor	**0.30**	**0.25, 0.37**	**0.23**	**0.19, 0.29**
Regular[a] consumption of alcohol (vs. No)	Yes	**1.21**	**1.07, 1.38**	**1.46**	**1.31, 1.63**
Smoking status (vs. Non-smokers)	Current smokers	0.80	0.73, 1.00	**0.76**	**0.65, 0.88**
	Beginning smokers	0.87	0.82, 1.17	**0.78**	**0.64, 0.94**
Regular[a] consumption of illicit drugs (vs. No)	Yes	**0.70**	**0.62, 0.80**	0.92	0.82, 1.15
School-level characteristics					
Schools deprivation index (vs. Most favoured public schools tertile)	Moderately favoured public schools tertile	0.96	0.81, 1.14	1.02	0.91, 1.13
	Less favoured public schools tertile	0.93	0.78, 1.10	1.03	0.92, 1.16
	Private schools	**1.29**	**1.08, 1.54**	**1.20**	**1.01, 1.40**
Number of parks or green spaces within 750 metres around the school (vs. High = 2 or more)	1 or 0 (low)	0.92	0.81, 1.05	**0.91**	**0.80, 0.98**
Cross level interactions					
Low number of parks or green spaces x Education level[b]		**0.88**	**0.79, 0.98**	1.05	0.96, 1.19
Random effects					
Variance components					
Level 2 variance: Estimate (SE)		0.27 (0.03)*		0.20 (0.02)*	
Intraclass correlation		7.59%		5.81%	

Abbreviations: HSQHSS, Quebec High School Student Health Survey; OR, Odds ratio (In our analysis, we ran multinomial logistic regression. Therefore, ORs are cumulative odds ratios, which are an average of the 3 logistic comparisons of LTPA levels: 'Sedentary' vs. others, 'Sedentary' or 'Slightly active' vs. 'Moderately active' or 'Highly active' and other vs 'Highly active'); 95% CI, 95% confidence interval. [a]: at least once a week for at least a month. [b]: Reference category: high number of parks or green spaces and lower education level[*]: p <0.001

© 2018 by the authors. Submitted for possible open access publication under the terms and conditions of the Creative Commons Attribution (CC BY) license (http://creativecommons.org/licenses/by/4.0/).
